# Supplementary material for: The Health and Nutritional Status of Children (10–18 years) Belonging to Food Insecure Households: The Korea National Health and Nutrition Examination Survey (2012–2019)
Source: Int J Environ Res Public Health. 2023 Aug 31;20(17):6695. doi: 10.3390/ijerph20176695 (PMC10487755; doi:10.3390/ijerph20176695)
Supplement: Supplementary file 1 [file ijerph-20-06695-s001.zip › ijerph-2497524-supplementary.pdf]

Table S1. Difference in Food Group Intake(%) & Individual Food intake(kcal/d) according to food insecurity status.

Food groups(%)	Boys			Girls		
	FS (n=1,650)	FI (n=221)	*p*	FS (n=1,500)	FI (n=184)	*p*
Food Group Intake/KDRI(%)[1)]						
Grains	118.6 ± 1.4	118.3 ± 4.4	0.952	111.2 ± 1.6	113.4 ± 3.9	0.592
Meat, fish, eggs, legumes	91.0 ± 2.0	89.7 ± 5.3	0.827	107.9 ± 2.6	93.6 ± 6.7	0.043
Vegetables	59.8 ± 1.6	62.8 ± 3.7	0.434	56.0 ± 1.3	55.6 ± 3.5	0.925
Fruits	69.8 ± 2.9	68.6 ± 10.3	0.909	126.8 ± 5.1	164.2 ± 25.3	0.150
Milk and dairy products	93.9 ± 2.7	90.7 ± 8.1	0.701	84.8 ± 2.4	95.7 ± 10.1	0.295
Oils, nuts and sugar	63.8 ± 1.8	55.0 ± 4.0	0.048	75.9 ± 2.4	68.8 ± 7.2	0.353
Individual intake(kcal/d) of subdivide of foods (KNANES)[2)]						
Cereals	1229.8 ± 11.6	1283.4 ± 26.4	0.062	948.7 ± 10.3	1003.5 ± 26.7	0.049
Potato and starches	94.8 ± 4.9	81.0 ± 12.4	0.308	86.5 ± 5.7	58.9 ± 11.7	0.040
Sugar and sweets	59.1 ± 3.7	50.2 ± 6.6	0.226	75.5 ± 4.8	77.0 ± 12.0	0.905
Legume and its products	55.4 ± 2.6	69.7 ± 11.4	0.217	41.4 ± 2.3	49.3 ± 7.7	0.335
Seeds and its products	13.8 ± 1.1	10.2 ± 1.5	0.053	14.7 ± 2.0	12.2 ± 3.0	0.482
Vegetables	65.5 ± 1.6	68.2 ± 3.5	0.474	50.9 ± 1.2	50.4 ± 3.0	0.876
Mushrooms	4.4 ± 0.5	4.5 ± 0.8	0.888	3.3 ± 0.4	3.1 ± 0.8	0.872
Fruits	104.6 ± 4.6	104.9 ± 13.7	0.982	103.0 ± 4.2	138.3 ± 18.9	0.072
Seaweeds	9.0 ± 0.6	10.6 ± 1.3	0.257	8.2 ± 0.7	11.6 ± 1.6	0.046
Oil and fat(plant)	88.4 ± 2.6	93.7 ± 6.8	0.466	71.3 ± 2.6	60.7 ± 6.0	0.102
Meats and its products	361.2 ± 9.5	350.6 ± 26.2	0.701	268.9 ± 7.5	255.9 ± 20.7	0.558
Eggs	75.9 ± 2.7	82.9 ± 6.7	0.337	65.6 ± 2.3	66.5 ± 6.0	0.889
Fish and shellfishes	63.8 ± 3.0	72.5 ± 8.8	0.338	57.9 ± 2.5	38.6 ± 4.7	<0.001
Milks and dairy products	234.5 ± 6.6	229.1 ± 20.1	0.800	212.9 ± 6.0	232.5 ± 23.2	0.414
Oils and fat(animal)	27.0 ± 2.7	33.1 ± 6.7	0.375	22.8 ± 1.9	23.5 ± 5.9	0.904
Beverages	163.5 ± 6.4	138.3 ± 16.9	0.170	135.7 ± 6.2	134.1 ± 24.1	0.948

Mean ± SE, P values are determined by student's t-test.

1) The average percentage ratio of Food group intake per KDRI recommended food group intake

2) Calorie intake(kcal/d) of individual foods except 4 foods (seasoning, other plant, other animal, alcohol) among 22 foods subdivided by KNANES.

FS: Food security group; FI: Food insecurity group, defined as a score of ≥3 according to the 18-item household food security assessment questionnaire.
